# Competition promotes the persistence of populations in ecosystems

**DOI:** 10.1038/srep30477

**Published:** 2016-07-27

**Authors:** Tao Wang, Jinqiao Duan, Tong Liu

**Affiliations:** 1 College of Science, Shihezi University, Shihezi, Xinjiang 832003, P. R. China; 2 Department of Applied Mathematics, Illinois Institute of Technology, Chicago, IL 60616, USA; 3 Center for Mathematical Sciences, Huazhong University of Science and Technology, Wuhan, 430074, P. R. China; 4 College of Life Science, Shihezi University, Shihezi, Xinjiang 832003, P. R. China

## Abstract

Competition is one of the most common form in ecological systems, which plays important roles in population dynamics. However, the influences of competition on persistence of populations remain unclear when space effect is included. In this paper, we investigated a predator-prey model with competition and spatial diffusion. Based on pattern formations and time series of populations, we found that competitions induce the persistence of populations, which denies *competitive exclusion principle*. Moreover, we testify the robustness of these effects. Our results also suggest that space may lead to the emergence of new phenomenon in ecosystems.

Ecological system is an important part of the real world, and populations constitute the ecological system^1^,^2^,^3^. Consequently, study on persistence of populations is one of the most important research fields of ecosystem^4^,^5^. Previous work showed that different factors have influences on the persistence of populations such as spatial motion^6^,^7^, time delay^8^,^9^ and random environments^10^,^11^.

Competition behaviors widely exist in the evolutions of populations, which have great effects on the persistence of the populations. One of the most important rules in competition systems is *competitive exclusion principle*^12^,^13^,^14^,^15^. From biological point of view, this principle suggests that two competitive species can not survive at the same time or in the same niche. In other words, it implies that competition behaviors play negative roles in the persistence of the populations.

Pattern formation in ecosystems is one of the central problems of the natural, social, and technological sciences^16^,^17^,^18^,^19^,^20^,^21^, which can reveal the distribution of the populations and provide useful information for protection of the population diversity. In recent ten years, pattern transition in ecosystems has being received more and more attention, including the mechanisms, ecosystem functions and so on^22^,^23^,^24^,^25^,^26^. In previous work^27^,^28^,^29^, it was found that competition may induce the populations to exhibit rich pattern structures, including mixed state of spotted and stripe pattern, labyrinth pattern and spotted pattern.

In this paper, we want to check whether competition behaviors have adverse impact in the persistence of the populations when space is included. In particular, we want to show the functions of pattern transition on the stability of the populations systems. The paper is organized as follows. We firstly show the pattern formation of the populations. Moreover, we reveal that competitions induce the persistence of populations. Additionally, we check robustness of persistence induced by competition.

## Results

In this part, we show our results based on a predator-prey model in reaction diffusion form (see Method section). In the first step, we display the spatial patterns in two cases and reveal the difference in these two cases. In the following step, we demonstrated competition would induce the persistence of populations. Finally, we revealed that the finding that persistence induced by competition is widely applicable in ecosystems.

### Spatial patterns of populations

Pattern structures may provide some important information on the state of the populations including persistence or extinction. Spatial patterns in different types imply different biological meanings. Our simulations employ the zero-flux boundary conditions with a system size of 200 × 200 space units. Time step is Δ*t* = 0.0001 and space step is Δ*h* = 1. And the initial density distributions are random spatial distributions of the predator and prey populations. During the simulations, different types of dynamics are obtained and the distributions of predator and prey are always of the similar type. As a result, we can restrict our analysis of pattern formation to one distribution (in this paper, we show the spatial pattern of predator populations).

**Figure 1:**
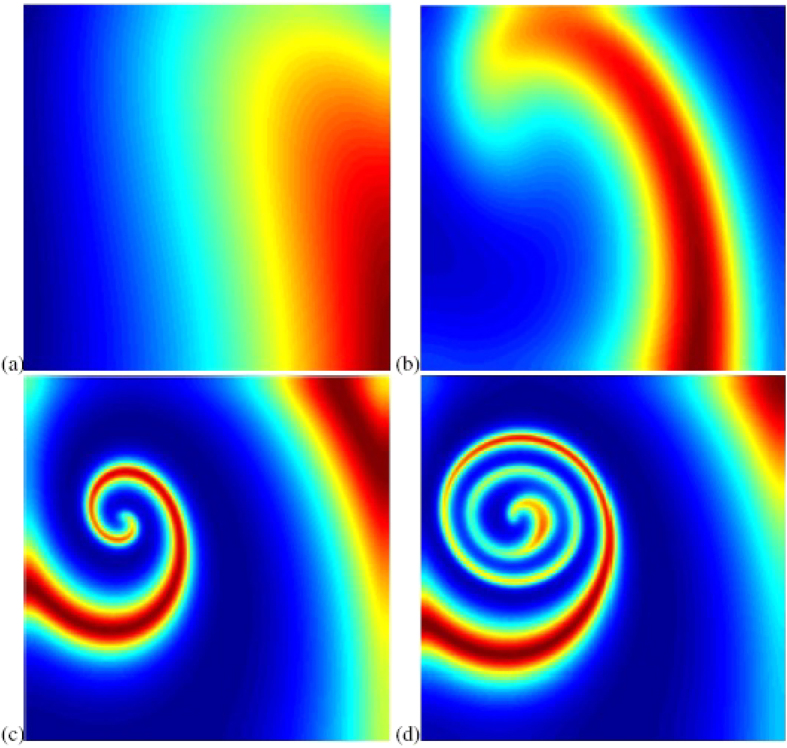
Spatial pattern of predator populations as time increases. This figure indicates that spiral wave can emerge if there is no competition in predator populations. (**a**) *t* = 100; (**b**) *t* = 200; (**c**) *t* = 500; (**d**) *t* = 1000. Prey populations have the similar pattern structures.

In order to declare our results, we show the spatial patterns of populations in two cases: (i) systems (7)-(8) without competition; (ii) systems (7)-(8) with competition. We perform a series of numerical simulations of the spatially extended systems (7)-(8) in two-dimensional spaces, and the dynamical behavior are represented by figures. In the first step, we show the spatial pattern of predator populations in systems (7)-(8) without competition (see Fig. 1) and the parameter sets are *a* = 1.3, *b* = 0.2, *l* = 1.1, *m* = 0.09 and *e* = 0. From Fig. 1, we can see that regular spiral waves emerge after the perturbation of the stationary solutions of the spatially homogeneous systems (7)-(8). After a long time, the spiral waves do not break up and stay stable in the two-dimensional space.

**Figure 2:**
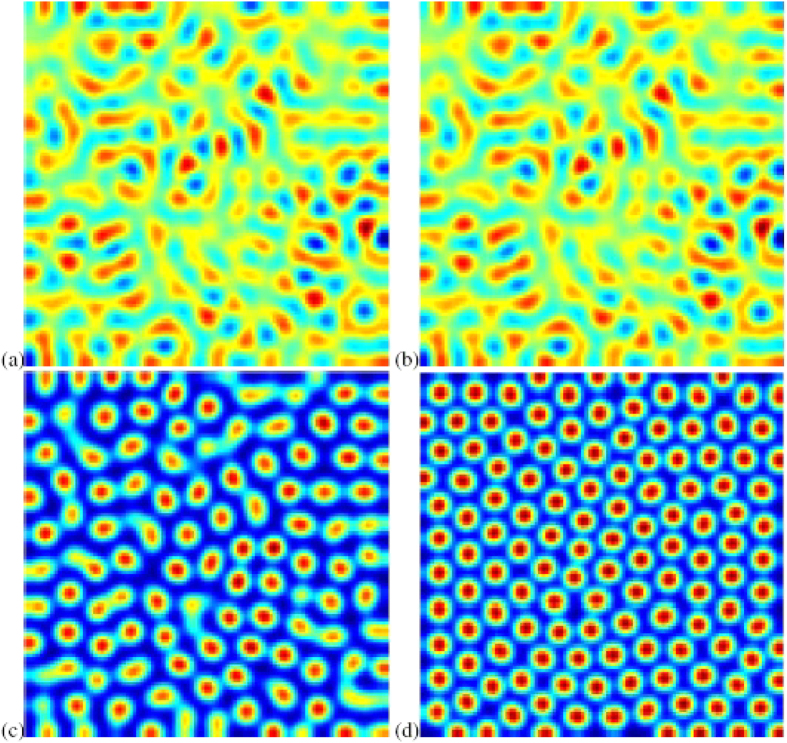
Spatial pattern of predator populations as time increases. This figure indicates that stationary pattern in spot form can appear if competition is considered in predator populations. (**a**) *t* = 100; (**b**) *t* = 200; (**c**) *t* = 300; (**d**) *t* = 1000. Prey populations have the similar pattern structures.

In the following step, we show the evolution of the spatial pattern of predator populations with competition at *t* = 100, 200, 300 and 1000 in Fig. 2 with *e* = 0.5. After the random conditions, spotted spatial patterns with different radius emerge. After some iterations, regular spots with the same radius prevail over the whole domain and the dynamics of the system does not undergo any further changes. Compared Fig. 1 with Fig. 2, one can conclude that competition in predator populations induces the pattern transition from no-stationary patterns (spiral waves) to stationary patterns (spotted patterns).

### Competitions induce the persistence of populations

**Figure 3:**
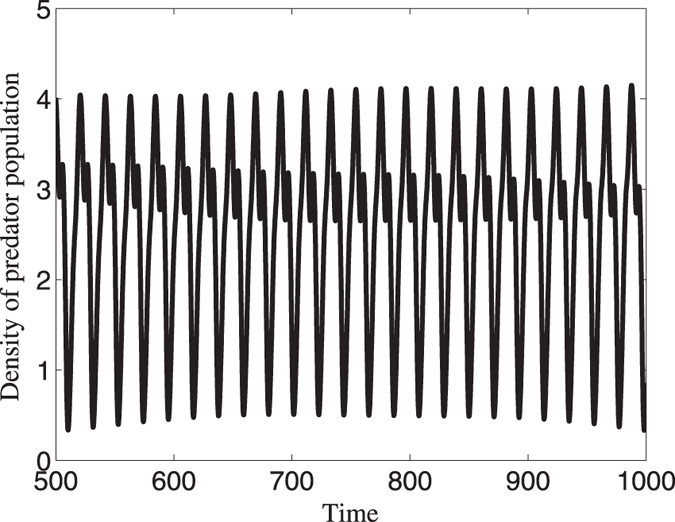
Time series of predator population when there is no competition. This figure shows that predator population has an apparent oscillatory behavior and may be in danger of disappearing.

**Figure 4:**
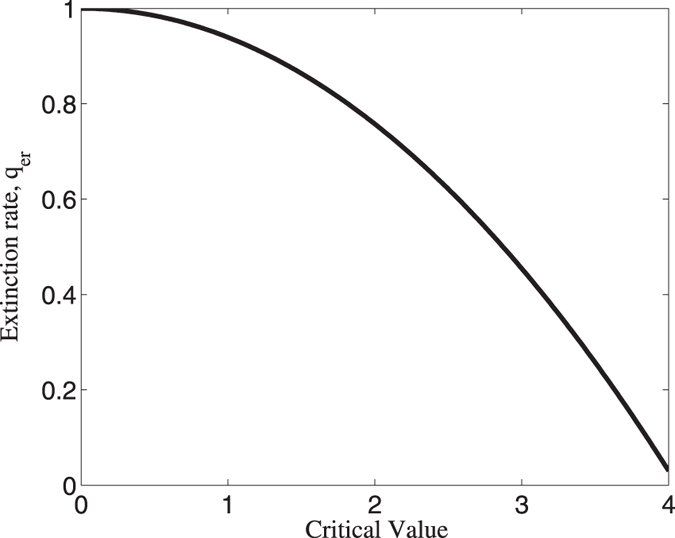
Extinction rate of predator population as a function of critical value. This figure implies that if the population under strong disturbance (i.e. critical value is small), then the population will tend to be extinct.

To see the effects of competition on the persistence of populations, we use time series analysis to show our results. In Fig. 3, we present the average density of predator population when there is no competition. For a long time, the density will exhibit a periodic behavior. However, the minimum value is too small and the predator population may suffer a high risk of extinction. In the real world, populations live in the natural environment and thus they are strongly influenced by abiotic factors such as weather and climatic conditions^30^,^31^. Under these circumstances, populations will be induced to be extinct if their density is smaller than a critical value. To intuitively represent the extinction risk, we give a quantity to measure the extinction rate:


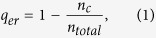


where *n*_*c*_ means the number of points when predator density is smaller than the critical density, and *n*_*total*_ is the total number points we employed. In Fig. 4, we found that the population may extinct if critical value is small. That is to say, if the population has spiral wave, then it is apt to die out.

**Figure 5:**
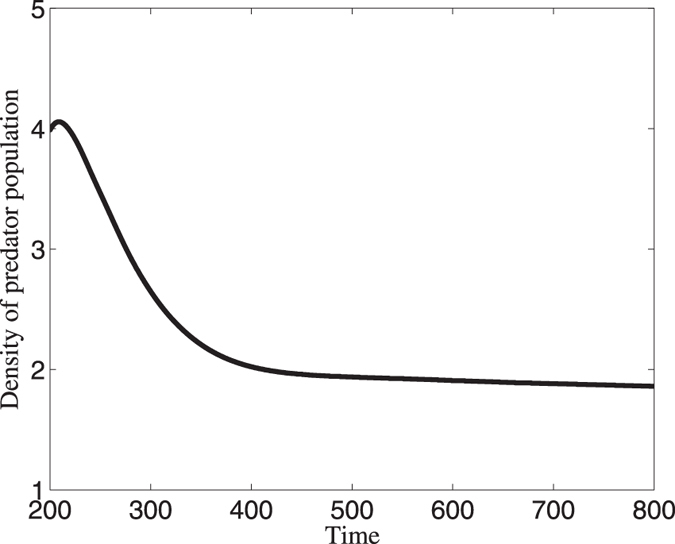
Time series of predator population when competition is combined in the systems (7)-(8). This figure shows that predator population will tend to a stable state as time is long enough.

When there is competition in predator population, the density of predator population will keep stable for a long time which is shown in Fig. 5. From this figure, we can find that the time series of the spatially averaged values of the population density shows that in the first intervals these values change fast as time increases. One can see that at *t* ≈ 350, the density of predator population reaches a constant value and the value increases much slower. As time further increases, the predator density has almost completely evolved and reaches its stable state. Based on time series analysis, we can draw a conclusion that competition may induce the persistence of populations.

### Robustness of persistence induced by competition

In the above part, we focused our attentions on the deterministic predator-prey system. We want to check that whether persistence of populations induced by competition can be found in stochastic environments. By numerical results on systems (18)-(19) (see Method section), we find that wave pattern can also be obtained if competition is not considered. Moreover, if critical value is small, then the populations may extinct with high rate. However, when competition is taken into account, then the population will go towards to stable state. Additionally, we further checked that in predator-prey systems with Holling type (such as Holling-II and III), competition can also promote the persistence of populations.

## Discussion

In this paper, we investigated a predator-prey model with competition and spatial diffusion. It was found that competition may induce the persistence of populations. In the previous work, the studies on two competitive species showed that the species can not survive at the same time or in the same niche^12^,^13^,^14^,^15^. Our results suggest that *competitive exclusion principle* does not hold when space is included, which well extend the findings in the fields of population dynamics. At the same time, our results imply that space may cause the emergence of new phenomenon and thus space should not be ignored in the investigations of ecosystems.

It should be noted that our results are obtained based on a predator-prey model in the reaction-diffusion form. In this sense, we need to check that whether our results still hold if predator-prey models in other forms, such as cellular automata^32^. What is more, we also need to use the real data to verify our conclusions which will be well discussed in the future study.

For spatial diseases, they may also exhibit pattern transition from no-stationary patterns to stationary patterns, which suggests that the disease may persist as an endemic state^6^,^16^. In this case, we need to take measures to control the diseases. That is to say, the results obtained in this paper are widely applicable from ecosystems to epidemiology.

## Method

### A predator-prey model

We give three principal rules on our model:

(1) In the absence of predator populations, prey populations grow logistically with intrinsic rate *r* and carrying capacity *K*;

(2) Functional response depends on the densities of both prey (*U*) and predators (*V*) populations:


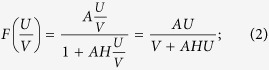


(3) Competitions only occurs in predator populations.

Based on the above rules, we obtain a predator-prey system with a ratio-dependent functional response and competitions which is as follows^33^,^34^,^35^:


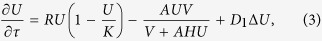



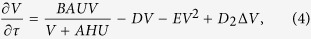


where *U*, *V* are prey and predator density, respectively. All parameters are positive constants, *R* stands for maximal growth rate of the prey, *K* carrying capacity, *A* capture rate, *B* conversion efficiency, *D* predator death rate, *H* handling time, and *E* competition rate. Δ = ∂^2^/∂*x*^2^ + ∂^2^/∂*y*^2^ is the usual Laplacian operator in two-dimensional space and *D*_1_, *D*_2_ are prey and predator diffusion coefficients.

For sake of calculations, taking


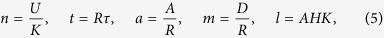



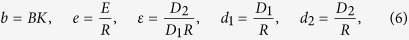


we arrive at the following equations containing dimensionless quantities (*V* is replaced by *p*):


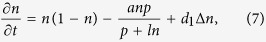



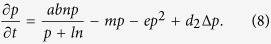


### Emergence of spatial patterns

Denote E* = (n*, p*) as the positive equilibria of systems (7)-(8). By linear analysis, we have the linear equations of systems (7)-(8):


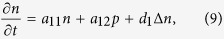



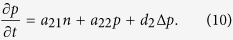


Following the methods in refs 36 and 37, we let:


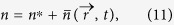


and


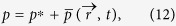


into the systems (7)-(8) and assume 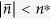 and 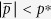. The initial conditions are assumed as: 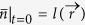 and 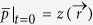, where the functions 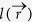 and 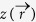 decay rapidly for 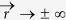. Following the standard approach, we perform a Laplace transformation of the linearized equations over the two independent variables 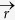 and *t*. For 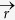 we use the so-called two-sided version of the transformation. The relations for the forward and backward transforms are


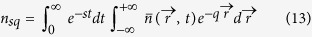


and


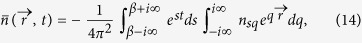


where *s* and *q* are complex variables. After this transformation, the kinetic equations read


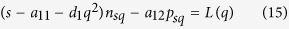


and


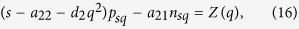


where *L*(*q*) and *Z*(*q*) are the transforms of 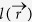 and 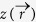. As a result, we obtain the denominator:


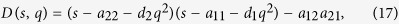


The condition for a spatial mode *q* (in one- or two-dimensional space) to be unstable and thus grow into spatial patterns is that Re(s) > 0.

### A predator-prey model with stochastic factors

When combined with noise term, the original spatially extended model (7)-(8) is written as the following system:


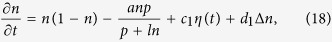



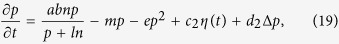


where *η*(*r*, *t*) is the noise term introduced additively in space and time, which is the Ornstein-Uhlenbech process that obeys the following stochastic partial differential equation^38^,^39^:


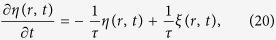


where *ξ*(*r*, *t*) is a Gaussian white noise with zero mean and correlation,


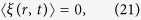



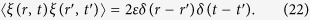


The colored noise *η*(*r*, *t*), which is temporally correlated and white in space, satisfies


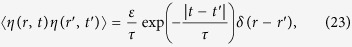


where *τ* controls the temporal correlation, and *ε* measures the noise intensity.

## Additional Information

**How to cite this article**: Wang, T. *et al*. Competition promotes the persistence of populations in ecosystems. *Sci. Rep.* **6**, 30477; doi: 10.1038/srep30477 (2016).
